# Breeding for Higher Yields of Wheat and Rice through Modifying Nitrogen Metabolism

**DOI:** 10.3390/plants12010085

**Published:** 2022-12-23

**Authors:** Pornpipat Kasemsap, Arnold J. Bloom

**Affiliations:** Department of Plant Sciences, University of California at Davis, Mailstop 3, Davis, CA 95616, USA

**Keywords:** cereal, biomass, NUE, yield component, nitrate, ammonium, adaptation

## Abstract

Wheat and rice produce nutritious grains that provide 32% of the protein in the human diet globally. Here, we examine how genetic modifications to improve assimilation of the inorganic nitrogen forms ammonium and nitrate into protein influence grain yield of these crops. Successful breeding for modified nitrogen metabolism has focused on genes that coordinate nitrogen and carbon metabolism, including those that regulate tillering, heading date, and ammonium assimilation. Gaps in our current understanding include (1) species differences among candidate genes in nitrogen metabolism pathways, (2) the extent to which relative abundance of these nitrogen forms across natural soil environments shape crop responses, and (3) natural variation and genetic architecture of nitrogen-mediated yield improvement. Despite extensive research on the genetics of nitrogen metabolism since the rise of synthetic fertilizers, only a few projects targeting nitrogen pathways have resulted in development of cultivars with higher yields. To continue improving grain yield and quality, breeding strategies need to focus concurrently on both carbon and nitrogen assimilation and consider manipulating genes with smaller effects or that underlie regulatory networks as well as genes directly associated with nitrogen metabolism.

## 1. Introduction

Balancing crop nitrogen and carbon status under changing environmental conditions is essential for sustaining agricultural productivity and food security. Nitrogen constitutes 1 to 2% of plant dry biomass, yet plants allocate a disproportionate amount of their energy to convert inorganic nitrogen forms, especially nitrate (NO_3_^−^) and ammonium (NH_4_^+^), into organic compounds [1]. As much as 50% of total plant protein may be ribulose-l,5-bisphosphate carboxylase/oxygenase (rubisco), the enzyme that initiates plant assimilation of CO_2_ into organic carbon through C_3_ carbon fixation [2]. Consequently, plant organic nitrogen and organic carbon are inextricably linked [3]. Rice (*Oryza sativa* L.) breeding has succeeded in increasing both grain yield and grain protein concentration in recent decades [4]. In contrast, long-term wheat (*Triticum aestivum* L.) breeding has achieved incremental biomass yield gain, but at the loss of grain protein content over time [5]. Plant breeders therefore actively seek to achieve two sometimes opposing goals, maximization both food productivity and quality.

Articles about breeding strategies to improve yield often discuss crop ideotype, outlining and dissecting desirable traits with the potential to achieve the highest theoretical yield or most rapid progress in genetic gains (for example, [6,7,8,9,10,11]). This review offers commentary presented in four sections: Process, Progress, Prospects and Puzzles. First, we briefly discuss crop inorganic nitrogen uptake, assimilation, and mobilization, topics for which a plethora of reviews already exist (for example, [12,13]). Second, we evaluate recent successful breeding endeavors involving genes within the nitrogen pathways that improve yield, using the framework of yield component analyses. Third, we present key trends among 40 validated genes that enhance crop yield. Highlighted are genes that influence tiller number, flowering time, and NH_4_^+^ assimilation. Lastly, we conclude by identifying areas for further research such as homologs across species, responses to different inorganic nitrogen forms, and complexities of natural variation and epistasis.

## 2. Process—Reaping What We Sow: How Soil Nitrogen Makes Its Way onto Our Plates

Plants acquire most of their nitrogen, both organic and inorganic forms, from soil, but reliance on each form varies greatly over time, with location, and under different environmental conditions [14]. Soil microorganisms mineralize organic nitrogen into NH_4_^+^, which subsequently becomes oxidized into NO_3_^−^ through nitrification [15]. Plants compete with soil microbes for NH_4_^+^, a form which also serves as a crucial microbial energy source [16]. In temperate aerobic agricultural soils, microbial activities rapidly convert most soil nitrogen into NO_3_^−^, and so NO_3_^−^ remains the dominant soil inorganic nitrogen compound available to crops [16,17].

Plant nitrogen acquisition relies on a well-coordinated network of transporters [13]. Nitrate transporters are among the most extensively studied groups of proteins and include low and high affinity systems that cover a large range of concentrations in soil; they also have additional functions beyond NO_3_^−^ transport [4]. Ammonium transporters are considered high affinity systems because they operate under low NH_4_^+^ concentrations [4]. The primary inorganic nitrogen assimilation pathway involves several reactions: nitrate reductase (NR) catalyzes NO_3_^−^ reduction into nitrite (NO_2_^−^), nitrite reductase (NiR) catalyzes nitrite (NO_2_^−^) reduction into ammonium (NH_4_^+^), and the concurrent actions of glutamine synthetase/glutamate synthase (GS/GOGAT) catalyze the incorporation of NH_4_^+^ into amino acids [18]. The resulting organic nitrogen compounds are transported, remobilized, and re-assimilated in different organs according to sink demand as a plant develops [4]. As plants mature and reach a reproductive stage, nitrogen compounds that have accumulated throughout vegetative stages are directed toward seeds, the organs vital to species survival and the harvestable part for most crops [4].

Our major focus here is wheat and rice for multiple reasons. First, these crops are the two top sources of plant protein that we consume daily according to the Food and Agriculture Organization of the United Nations (FAO) [19] (Table 1). Relative reliance on these two crops as a major protein source varies across geographical regions. Wheat contribution to human protein intake is dominant in Northern Africa (38%), Central Asia (38%), Southern Asia (26%), Western Asia (39%), and Europe (22–29%). Rice prevails in South-eastern Asia (34%), Southern Asia (21%), and Micronesia (18%). Second, wheat and rice are both C_3_ plants belonging to the Poaceae family. Such relatedness may facilitate the transfer of knowledge between these two closely related species, although the genome of hexaploid wheat is 40 times larger than that of rice [20]. Third, as model species and major food crops, they both have been the subject of extensive research extending over a broad range of production areas across diverse environmental conditions worldwide [21]. Lastly, under current cultivation practices, wheat and rice may have adapted to different habitats [22], especially to distinct forms of inorganic nitrogen. Wheat is grown in aerobic soils, dominated by NO_3_^−^, whereas rice is grown usually under hypoxic conditions with a relatively high NH_4_^+^ presence in the root zone. Understanding how major food crops adapt to different forms of nitrogen should highlight nutrient management strategies to improve grain yield and quality. Three major components contribute to yield of small grain crops: number of tillers, number of grains per tiller (or grains per spike), and grain weight [23]. Number of grains per tiller may be further divided into number of panicles (or spikelets) and number of grains per panicle (or spikelet). Whereas tiller development can be influenced significantly by changes in the environment, grain characteristics are highly heritable [23,24]. Grain number and grain yield are positively correlated with crop nitrogen content [21]. Crops absorption of NO_3_^−^ and NH_4_^+^ from soils and assimilation into organic forms reaches a peak at anthesis [25]. During grain production, plants remobilize stored organic nitrogen compounds and translocate them to seeds [26]. Nitrogen supply from before planting until anthesis is more strongly related to vegetative growth and yield potential, while nitrogen application post-anthesis is more strongly related to improved protein content and grain quality [25,27]. Photosynthesis, a process in which nitrogen-rich compounds play a major role, contributes biomass to fill in grain weight [21]. In other words, nitrogen is fundamental to all processes that determine final grain yield [11]. Therefore, optimizing nitrogen acquisition throughout crop development is crucial for attaining maximum yield potential.

## 3. Progress—Common Breeding Strategies Are Limited to Regulating Expression of Few Genes 

Plant breeders achieve genetic gain in breeding populations over generations by selecting and retaining genetic materials with targeted characteristics and superior performance. A more thorough understanding of the molecular biology and genetic basis of specific traits facilitates the rapid development of more desirable genotypes, especially for traits that are controlled by a single or few loci with large effects. Yet, improving complex traits like yield and nitrogen responses remains challenging.

Will breeding for improved nitrogen uptake and assimilation also increase yield? While yield improvement can arise from factors affecting yield components besides increased nitrogen use efficiency (NUE), breeding for this trait should lead to increased biomass production and grain yield [28,29]. Nonetheless, breeding programs for yield seldom monitor nitrogen responses [30,31,32,33], and modern cultivars with higher yield demonstrate little improvement in NUE [34].

The genetic basis underlying desirable phenotypes for grain yield and quality often remain obscure, despite genetic gains through selection. For example, in Green Revolution varieties, the genetic variants and mechanisms responsible for the short stature and increased harvest index that underpin the yield boost were identified only several decades after the release of improved cultivars [35]. In rice, the recessive loss-of-function mutation of *Semi-Dwarf 1* (*SD1)* impairs an oxidase enzyme in the synthesis pathway of gibberellin, a key hormone promoting height, whereas in Green Revolution-derived varieties of wheat, mutations of *Reduced Height 1 (RHT-1)* encode modified proteins that also diminish height, but are insensitive to gibberellin-induced degradation [36,37,38,39]. Dwarfing genes improve yield through several mechanisms that act in concert to both diminish height and significantly increase biomass partitioning to the grain [36]. High harvest index, the fraction of biomass allocated to harvestable organs, is known to be strongly associated with high crop nitrogen status [40]. Unfortunately, many Green Revolution phenotypes, regardless of the mechanisms responsible for decreased gibberellin responses, also limit crop responses to nitrogen [41,42]. Plants with a dwarfing gene often have slower nitrogen uptake [42] and nitrogen accumulation relative to dry matter accumulation after anthesis, thereby decreasing NUE on a grain biomass basis in the field [41]. Insensitivity to increased nitrogen supply may be beneficial because the absence of nitrogen-promoted stem elongation makes plants more resistant to lodging [42], although at the high cost of requiring additional nitrogen fertilizer to maintain adequate yield. This case study from Green Revolution varieties underscores the challenge of improving yield through modifying nitrogen metabolism.

Generally, attempts to improve yield also alter rates or paths of metabolite production [43]. In particular, the enhanced harvest index of widely grown Green Revolution varieties diverts more biomass into harvestable grains. Nonetheless, assuming we have not reached the limits of biomass production, we may coordinate source vs. sink balance and continue to allocate additional crop assimilation of carbon and other nutrients toward yield [44]. Although efforts to increase crop source strength in terms of light capturing efficiency have been long underway [45], this goal seems elusive unless we address water and nutrient limitations [46,47,48]. Greater emphasis on enhancing nitrogen accumulation upon which biomass production depends may prove more effective in increasing yield in the near future [46,48]. With more extensive knowledge about the genetics of the underlying traits and advanced breeding tools, we could perhaps make even faster progress if we target both enhanced carbon and nitrogen assimilation concurrently.

An extensive body of literature is now available about the major transporters and enzymes associated with nitrogen assimilation and remobilization throughout crop growth cycles [4,13,30,49]. Characterized and cloned are key genes that govern metabolic pathways, but successful breeding applications for yield improvement that involve these genes are few, especially those that have reached the stage of commercial field trials [12]. 

Here, we have tabulated 40 genes that influence nitrogen metabolic pathways and improve grain yield (Table 2). Among these, regulation of gene expression seems to be the most successful approach for translating improved nitrogen metabolism into higher yields. Overexpression of genes [50] is the most common approach. Less common is knocking out [51] or silencing the genes of interest with small interfering RNA (RNAi) [52] or Clustered Regularly Interspaced Short Palindromic Repeats (CRISPR/Cas9) [53] that precisely targets specific genomic regions. Relatively few studies have employed conventional breeding methods and, thus, have avoided transgenic means for introgression or incorporation of a functional allele into a breeding population. Progress in rice overall has been more rapid than in wheat [54]. Below are four different categories of genes involved in nitrogen metabolism that recent breeding efforts have manipulated to improve yield.

### 3.1. Nitrogen Transporters (17 Genes)

Modern phylogenetic studies classify major families of nitrogen transporters in land plants based on their substrate: nitrate, ammonium, or peptides [99]. Most characterizations of transporters are in *Arabidopsis* and rice [100]; relatively limited information is available for transporters in wheat [30,101,102]. Nitrate transporters (11 genes) have received more attention and their potential for yield improvement have been evaluated more thoroughly than transporters of ammonium and organic nitrogen [13,33]. Perhaps this derives from NH_4_^+^ being a nitrogen source that only dominates in a few agricultural production systems and from the ability of its counterpart NH3 to move freely through membranes following electrochemical gradients [1]. Ammonium transporters (2 genes) may also prove to be more elusive as a target for yield improvement because of the potential for toxicity from excessive accumulation of free NH_4_^+^ in tissues as discussed below [33,103]. Nonetheless, coupling NH_4_^+^ uptake with assimilation by concurrent modification of *AMT1;2* and *GOGAT* can drive yield improvement [69].

Modification of amino acid transporters (*AAP*, 4 genes), despite receiving less attention than that of NO_3_^−^ transporters [12], is another effective strategy for increasing grain yield. These transporters, in contrast to those that transport NO_3_^−^ or NH_4_^+^, are key players in remobilizing assimilated organic nitrogen compounds, although their exact functions remain largely unknown in cereals [104]. Organic nitrogen transport within plants directly relates to grain nutritional quality at maturity [26]. Of particular interest is that variation in the promoter regions across germplasm suggest tight expression regulation and local adaptation that may help plants cope with fluctuations in soil nitrogen gradients [75,76,77].

Overall, while we have some understanding of how transporters contribute to uptake and transport of each nitrogen form across membranes and might improve plant nitrogen acquisition, modification of these transporters has had limited success in crop yield enhancement [33].

### 3.2. Nitrogen Assimilatory Enzymes (5 Genes)

Assimilation of inorganic into organic nitrogen in plants is well-regulated at transcriptional, translational, and post-translational levels [105,106]. The enzymes GS and GOGAT are central to nitrogen metabolism, but attempts to alter yield by modifying genes coding for these enzymes have achieved only little success. Previous modification to *GS1* increased nitrogen partitioning to grain and nitrogen harvest index, but not vegetative yield nor overall shoot nitrogen accumulation [107]. Failure to successfully modify *GS1* on its own may derive from the critical functions for which this gene is responsible [108]. By contrast, modifying *GS2* can lead to wheat yield improvement in stressful environments [71]. Alteration to *GOGAT* expression to boost yield was only achieved through changing expression levels of transcription factors upstream of the enzyme (see discussion below). Thus, successful breeding applications coupled *GOGAT* with changes to ammonium transporter AMT1;2 [69] or simultaneously modulated *GS1* and *GS2* [70]. Because *GS1* and *GS2* are involved in crop growth at different developmental stages [109,110], selecting the appropriate developmental time to express each of these enzymes was critical for a positive result [70].

### 3.3. Nodule INception-like Proteins That Sense NO_3_^−^ and Regulate Downstream Genes (3 Genes)

Legumes when associated with certain bacteria can generate organic nitrogen from dinitrogen gas N_2_ in air, a process named symbiotic nitrogen fixation [111]. *Nodule INception (NIN)* genes govern legume root nodule initiation and symbiotic nitrogen fixation [112]. *NIN-like proteins (NLPs)* that are homologs to *NINs* found in non-leguminous crops have critical roles in regulating nitrogen signaling and downstream genes within nitrogen metabolism [113].

Multiple highly conserved *NLPs* are found in *Arabidopsis* [112], wheat [114], and rice [112]. *Arabidopsis NLPs* function as transcription factors, and *NLP7* also serves as a biosensor responsive to intracellular NO_3_^−^ supply [115]. Rice *NLPs* generally serve as activators that control expression of nitrogen responsive genes. For example, *NLP4* regulates expression of genes underlying key enzymes in nitrogen assimilation pathways [80], thereby affecting activities of *NiR* [81] and *NR* [116]. Some *NLPs* also shows nitrogen form-specific responses with NO_3_^−^ being the major form to which rice *NLPs* are most responsive. While either NO_3_^−^ or NH_4_^+^ can trigger expression of *NLP3*, only NO_3_^−^ induces its nuclear retention [79]. Overexpression of these *NLPs* in rice stimulate yield, whereas reduced expression of *NLPs* inhibits growth. To date, yield improvements of wheat from modifications of *NLPs* are lacking, although nitrogen starvation upregulates *NLP7* [114] and *NLP4* [117].

### 3.4. Transcriptional Factors and microRNA That Regulate Other Genes (15 Genes)

Transcriptional factors bind to the promoter of target genes to regulate downstream gene functions [118]. System biology is steadily clarifying how a large network of transcription factors regulate nitrogen metabolism and how key transcription factors control expression of multiple proteins in the pathways simultaneously [119]. Whereas modifying expression of individual transporters and enzymes has had only modest success in improving crop performance, altering transcription factors that orchestrate simultaneously systematic changes in multiple nitrogen-related genes may have profound effects on biomass accumulation and grain quality. For example, overexpressing rice *DREB1C*, which regulates nitrogen assimilation genes, increased nitrogen assimilation and photosynthesis significantly, resulting in increased grain number, grain weight, and harvest index [95]. Together these changes resulted in 68.3% higher yield than wildtypes and in a 13 to 19 days earlier flowering time [95]. Light- and nitrogen-mediated *OsDREB1C* controlled over 9000 genome-wide putative binding sites, including five gene targets in the carbon and nitrogen metabolism pathways: *rubisco small subunit 3 (OsRBCS3)*, *OsNR2*, *nitrate transporter 2.4 (OsNRT2.4)*, *OsNRT1.1B*, and *flowering locus T-like 1 (OsFTL1).* Previous attempts to engineer several individual genes from this list never reached as high a yield gain as manipulating the transcriptional factor gene *OsDREB1C* alone. For instance, overexpression of transporters led to higher accumulation and efflux of excessive nitrogen because plants were not able to assimilate more nitrogen into protein [120]. 

Manually coordinating individual genes underlying nitrogen sources and sinks to complete a whole pathway therefore remains a challenge. Although we still have limited understanding about the regulation and function of transcription factors, modifying a single transcription factor appears more effective than manipulating individual genes and proteins in a pathway [121]. This highlights the complexity and tight regulation of nitrogen metabolism. As more genotypic and phenotypic data become available across diverse plant species, the roles of transcriptional factors in nitrogen metabolism should become clearer. Editing genetic networks, rather than individual candidate genes that regulate the balance between carbon and nitrogen metabolism may prove to be a more promising approach for increasing yield.

## 4. Prospects—Fine-Tuning Yield Component Responses to Transient Nitrogen Supply Can Maximize Yield

Successful manipulation of genes regulating nitrogen metabolism (Table 2) is contingent upon more advanced understanding of how nitrogen acquisition influences growth and vice versa. We now have a better understanding on how nitrogen, especially NO_3_^−^, drives hormonal and physiological changes underlying canopy architecture and development [122]. However, the influence nitrogen has on yield components and the tradeoffs among subcomponents are still uncertain. In this next section, we summarize recent findings, focusing on tiller number, flowering time, and NH_4_^+^ assimilation as key links between carbon and nitrogen metabolism and, therefore, highly relevant to nitrogen-driven yield improvement. 

### 4.1. Tiller Production Contributes to Higher Yields through Multiple Nitrogen-Mediated Signaling Pathways

Tiller number, a key determent of effective number of panicles that contribute to grain filling and grain yield, is the most responsive of all yield components to nitrogen [122]. Tiller number is a routinely measured yield component because its assessment is straightforward. Out of the 40 genes reported to improve yield in this review (Table 2), 19 genes are associated with higher tiller number. 

Increasing nitrogen supply generally increases tiller production [82] whereas limiting supply decreases tiller production [122]. Soil NH_4_^+^ concentration correlates linearly with tiller number in rice [123] and nitrogen fertilization levels explain 66% to 96% of the variation in tillering rate, which is significantly correlated with the final grain yield [23]. Similarly, increased nitrogen levels also boost tiller production in wheat [124]. Changing canopy architecture by optimizing nitrogen inputs and increasing tiller number per unit area thus enhances biomass source strength and grain yield in rice [125], and both yield and grain protein content in wheat [126].

Changes in tillering number derive from the interplay between multiple opposing nitrogen-mediated hormonal shifts [122]. High nitrogen availability induces cytokinins to increase tillering, but also induces auxins and strigolactones to inhibit tillering [127]. In rice, multiple amino acid transporters balance the opposite actions of auxins and cytokinins: *OsAAP1* and *OsAAP4* regulate auxin and cytokinin signaling [74,76], whereas *OsAAP5* only influences cellular cytokinin levels [77]. *microRNA393* (*OsmiR393*), in turn, lowers sensitivity to auxin signaling and increases tillering [128]. 

Feedback mechanisms between hormones and nitrogen ensure optimized developmental responses to fluctuating external nitrogen pools. As nitrogen supply increased, a negative feedback mechanism driven by *DNR1* reduced auxin functions to upregulate genes for tiller production and nitrogen metabolism, thereby repressing nitrogen uptake and assimilation as well as tiller production [94]. Conversely, a nitrogen shortage downregulated *DNR1*, promoting nitrogen acquisition and tiller development [94].

The complex balancing acts of gibberellin, which explain why Green Revolution plant varieties maintain lower height and high yield, but require high nitrogen fertilizer inputs, have been reviewed in great detail elsewhere [106]. In brief, gibberellin and its counterparts DELLA proteins, which are named after their conserved chain of amino acids D-E-L-L-A, have two fates. Under high nitrogen availability, gibberellin can either inhibit tiller development by degrading gibberellin’s downstream transcriptional factor *NGR5* protein or promote tiller production via a positive feedback mechanism driven by nitrogen itself to increase nitrogen assimilation and upregulate *NGR5,* which represses tiller inhibitory genes. Likewise, DELLAs may sustain tiller promotion by interfering with gibberellin-driven NGR5 destruction [82] or decrease nitrogen accumulation by downregulating nitrogen assimilation genes [42], thereby indirectly limiting nitrogen-driven tiller development. Because most Green Revolution-derived high-yielding cultivars already contain dwarfing genes conferring high DELLA abundance, breeders can further increase tiller production and yield even at low nitrogen levels by increasing NGR5 abundance directly, suggesting a potential decoupling of tillering from nitrogen supply [82].

Modification of transcriptional factors further enhances yield by tipping the balance of proteins and promoting nitrogen-driven tiller production. The coordination for carbon and nitrogen is systematically regulated by the transcription factor *GRF4* [82] and its upstream repressor MIR396 [94], both of which modulate nitrogen acquisition and growth via *DNR1* [94] and modulate nitrogen assimilation genes to counterbalance the inhibitory effects of DELLA [42]. Therefore, increased *GRF4* expression alters the balance of GRF4-DELLA, thus enhancing nitrogen assimilation, tiller development, and grain yield [42]. 

Nitrogen influence on tiller development via the brassinosteroid signaling pathway also remains an active area of research. High NO3^−^ levels decrease rice expression of *TCP19*, which represses *Dwarf and Low-Tillering (DLT)*, a gene involved in brassinosteroid signaling and tillering promotion, thereby inhibiting tiller bud outgrowth [83]. *OsTCP19* overexpression lines exhibit brassinosteroid-deficient phenotypes similar to *dlt* mutants [83]. In wheat, overexpression of *Dwarf4 (DWF4)*, which encodes a key enzyme in brassinosteroid synthesis, also increases both nitrogen assimilation and tiller number [129]. Furthermore, the proteins of rice *DLT* and *MONOCULM1 (MOC1),* which regulate tiller production, are both under control of *NGR5* [82].

Interestingly, there are tradeoffs among yield subcomponents. For example, not all yield improvement is associated with increased reproductive tiller number. In fact, fewer tillers is a key characteristic proposed as an ideal canopy architecture for high yields [130]. Mutants with loss-of-function *dnr1* or reduced DNR1 abundance develop fewer tillers, but increase auxin, accelerate nitrogen uptake, and exhibit higher yields [94]. In the case of *DREB1C* overexpression, transgenic rice plants with higher yields have fewer panicle numbers, but instead produce elongated panicles with increased grain weight and number of grains within each panicle [95]. Such coordination between source and sink components appear to shift if carbohydrate supplies increase because these transgenic plants also have higher photosynthetic rates and accumulate more biomass at heading stage. Additionally, reduced branching may also result from a shortened development period to be discussed in the next section. Altogether, regulations of nitrogen-mediated tiller development highlight the importance of evaluating all yield components that contribute to actual yield changes.

### 4.2. Optimized Flowering Time Maximizes Nitrogen and Carbon Assimilation in Agricultural Settings

Adjustments of flowering time or heading date is an evolutionary adaptation that maximizes seed yield and survivability over generations [131]. Flowering time optimized for each environment can enhance grain yield in staple food crops [132]. The transition from vegetative to reproductive developmental stages determines total nitrogen accumulation over the vegetative growth period [25,32] and shifts the emphasis of nitrogen metabolism to remobilization and reassimilation in maturing grains [26]. While photosynthesis per unit leaf area may remain unchanged, cumulative increases in leaf area, light interception, overall growth period, and vegetative biomass accumulation—all responsive to nitrogen inputs—may together increase yields [133]. Suboptimal or excess nitrogen supply often, respectively, accelerate or slow the transition to reproductive phase [134]. The precise extent to which nitrogen supply influences cereal flowering time, however, is uncertain [135].

The genetics underlying vernalization and photoperiod pathways in cereals are well-characterized [136], but their interactions with nitrogen remain an open question. Multiple genes regulate flowering time and its influence on grain yield [132]. Indeed, genes underlying developmental timing like *Photoperiod* (*Ppd*) and *Vernalization* (*Vrn*) appear to co-locate with Quantitative Trait Loci (QTL) associated with NUE [137], suggesting a potential connection with nitrogen metabolism. Several recent studies have identified genes with pleiotropic effects that change both nitrogen responsiveness and crop developmental timing via senescence and flowering time. These include *NPF6.3*, *GS2*, *Nhd1*, *Ghd7*, *ARE1*, *miR396* (Table 2). Specifically, transcription factors *Nhd1*, *Ghd7* and *DREB1C* have direct control on genes involved in determining heading date [86,90,95]. Connections of other candidate genes with developmental timing require further validation.

An appropriate flower timing is essential for avoiding stressful conditions and maximizing favorable conditions for seed production [131]. Most genes in Table 2 promote a longer growing season. Prolonged vegetative growth generally allows crops to accumulate and assimilate more nitrogen before a crop reaches maturity and senescence, potentially resulting in increased NUE and biomass accumulation. However, a longer growth season may also increase the chance of experiencing abiotic and biotic stresses [131]. Only *NPF6.3*, *DREB1C*, and *RDD1* accelerate a transition to the reproductive stage and still show a yield improvement [56,95,97]. For example, *OsDREB1C* significantly enhanced yield, despite a 2–3 weeks shorter growth period [95]. The ability to accumulate higher biomass under a shorter timeframe indicates a higher capacity for carbon and nitrogen assimilation. Nevertheless, early flowering time in rice with photo-insensitive alleles was previously shown to be associated with reduced grain filling, fewer panicles, and subsequently lower yield [138]. 

Developmental changes driven by variations in the growth environment determine the extent to which yields can be improved. Varying outcomes from different modifications may derive from the environmental interactions underlying nitrogen influence on growth and development. Late season tiller production may not produce a fertile fluorescence and thus contributes only to vegetative biomass production [35]. Tillers initiated early in the season also tend to have higher yields than late tillers [139]. High tiller production combined with longer maturation time generally contributes to higher rice yield [140]. For *OsDREB1C* modifications, overexpressing plants grown under long days and temperate climates flower about 50 to 70 days later and have higher yield improvement rates than plants under other experimental conditions [95]. Photoperiod pathways seem to be likely candidates that connect nitrogen responsiveness with flowering time, although no known mechanisms have been confirmed to date [134]. Understanding how carbon and nitrogen assimilation intersect and their environmental interaction in the context of crop developmental timing will be crucial in matching crop demands with resource supplies.

### 4.3. GOGAT as an Indirect Target—A Case Study from Editing Transcriptional Factors ARE1, Nhd1, and bZIP60

GOGAT, when coupled with GS, catalyze the assimilation of NH_4_^+^ into glutamate, an amino acid central to nitrogen and carbon metabolism [141]. Based on genetic map synteny, a meta-analysis of cereal QTL studies on NUE identified *GOGAT* as a candidate gene that is conserved among major food grain crops (rice, wheat, sorghum, maize) [137]. Although editing *GOGAT* directly has little influence on yield, coupling *GOGAT* with *AMT1;2* proved effective in enhancing yield [69]. Modifying transcription factors upstream of *GOGAT* also has been successful (Table 2): *ARE1* and *Nhd1* both suppress *Fd-GOGAT* [87,89,90], while *bZIP60* suppresses *NADH-GOGAT* [85]. 

Eliminating suppression of *GOGAT* via these transcription factors, improves yields significantly. Enhancement of *GOGAT* function seems to be a plausible approach for raising yield because glutamate links carbon and nitrogen metabolism [141]. The role of transcriptional factors suggests that we have yet to characterize additional players involved in the assimilation of NH_4_^+^ into organic nitrogen. Identification and modification of other pathways similar to *GS/GOGAT* -driven NH_4_^+^ assimilation, in that they influence both nitrogen acquisition and remobilization, may prove most effective for improving yields. 

## 5. Puzzles—Knowledge Gaps about Modifying Nitrogen Metabolism for Yield Improvement

The current body of literature proffers open questions that require further investigation. In particular, studies that compare homeologs across species, crop responses to different inorganic nitrogen forms, and quantitative genetics underlying crop adaptation to natural soil nitrogen gradients should accelerate yield improvement through modified nitrogen metabolism.

### 5.1. Differences among Homologs across Species Remain Elusive 

Comparative studies among species offer unique insights into finding related genes underlying desirable traits [142] such as for genes involved in C_4_ carbon fixation [95]. Nevertheless, transfer of successful breeding strategies across species remains challenging, even decades after fully characterizing most elements in nitrogen metabolism pathways [143]. 

To date, studies have identified more candidate genes and generated more breeding applications related to NUE in rice than in wheat [22,54] (Table 2). Translating insights from rice to wheat require herculean efforts, largely because of differences in genomic size and structure [22,144,145]. New mutant resources [146] and transgenic tools [147], however, increase the feasibility of characterizing candidate genes across a polyploid genome. Novel approaches like CRISPR-Cas9 system also further allows more precise editing of targeted loci of interest [148]. Even cross-species gene modifications such as transforming rice with wheat *TaGS1* have proved successful in enhancing rice yield [149].

Multiple yield-determining genes are shared among rice, wheat, maize, and barley [22,150]. Identification of orthologous genes offers an alternative to introducing foreign genetic materials. Here, we discuss three examples: *GOGAT*, *DREB1C* and *Ghd1*. First, *GOGAT* is well-conserved in rice, wheat, sorghum and maize [137]. Editing transcription factors regulating *GOGAT*, however, seems more effective than modifying individual genes on their own (see discussion above). Second, rice *OsDREB1C*, whose overexpression increased yields up to 68.3%, has an ortholog in wheat *TaDREB1C*, whose overexpression results in 22.6% more grain yield than wildtypes [95]; the reason for the large differences in yield enhancement among species is not yet understood. Third, *Ghd7* in rice and its ortholog *VRN2* in wheat [86] have a high potential to improve agricultural performance. Both genes are well-studied and control flowering time in their respective species [151,152,153]. To date, however, there is no clear evidence on how *VRN2* integrates signaling from nitrogen into regulation of flowering time in wheat. Given the promising yield enhancements attained with *Ghd7* in rice, *VRN2* might also provide major increases in wheat yields, but this is still unknown.

### 5.2. Insights on How Inorganic Nitrogen Forms Affect Crop Responses Are Lacking

Each form of inorganic nitrogen, NH_4_^+^ or NO_3_^−^, triggers specific crop responses [154]. In particular, an exposure to high concentration of soil NH_4_^+^ is generally toxic to most plants because root absorption of NH_4_^+^ may exceed the capacity of the plants to sequester the NH_4_^+^ in vacuoles or assimilate it into organic forms [103]. As free NH_4_^+^ accumulates within plant tissues, it can dissipate pH gradients through which mitochondrial and chloroplastic electron transport generate ATP [1]. To avoid such ill effects, plants generally assimilate NH_4_^+^ in roots and transport organic nitrogen compounds to other organs [103]. Optimizing root NH_4_^+^ accumulation and assimilation can enhance plant NH_4_^+^ tolerance and overall nitrogen acquisition [155]. By contrast, plants can store relatively large amounts of free NO_3_^−^ without ill effect [1], and it serves as major signaling molecules for a number of metabolic pathways [156]. 

Although the importance of each inorganic form as a nitrogen source in crop production is well established [157], information is still meager on how each form induces or suppresses expression of nitrogen responsive genes or how changes in these genes in turn affect uptake and assimilation of each form. For example, NO_3_^−^ transporter genes have a strong influence on NH_4_^+^ metabolism, and vice versa [158]. A more comprehensive understanding of these interactions would be crucial to designing and implementing more effective nitrogen fertilizer management strategies.

Relatively few studies compare responses to both form of inorganic nitrogen side by side, let alone evaluate the responses to a range of concentrations in diverse genetic materials. Although the model species *Arabidopsis* usually exhibited higher biomass and root production under NO_3_^−^ nutrition, this species showed a wide range of distinct phenotypic responses and gene expression pattern when receiving NO_3_^−^ or NH_4_^+^ as a sole nitrogen source [159]. Wheat growth under either form also demonstrated distinct accumulation and distribution patterns of other essential nutrients [160]. Nonetheless, we do not have sufficient information about the extent to which editing major genes in the nitrogen metabolism pathways changes responses to each inorganic form, and whether responses in wheat and rice are like those observed in *Arabidopsis*.

Most experiments to characterize genes reported in this review have only focused on a single nitrogen form or fail to designate the nitrogen form at all (Table 3). Detailed characterizations of individual nitrogen transporters may show that, not only are they responsible for uptake of both NO_3_^−^ and NH_4_^+^ (for example, *NRT 2.3b* [64]), but also their functions have expanded and co-evolved to interact with other biotic and abiotic factors [161,162]. For example, *NPF6.5* not only regulates NO_3_^−^ uptake, but is also associated with recruitment of root microbes involved in the synthesis of NH_4_^+^ [158]. Understanding balance in crop utilization of both inorganic nitrogen forms will help us improve our crop and fertilization management in response to changing environments [163].

Nitrogen interacts more strongly with carbon assimilation as nitrogen supplies limit crop responses to enriched atmospheric CO_2_ levels [173]. Meta-analyses demonstrate that nutritional quality of wheat and rice—especially protein and micronutrients such as iron and zinc—decline significantly under elevated CO_2_ levels [174]. Among several alternative explanations for the declining crop protein at elevated CO_2_ levels [175,176,177,178], direct inhibition of shoot nitrogen assimilation [179] is most consistent with observations under a wide range of experimental conditions [180,181,182]. 

Photorespiration provides energy for shoot NO_3_^−^ assimilation in C_3_ plants [3]. Photorespiration generates reductants when atmospheric CO_2_, but not light levels, limits photosynthesis and enables C_3_ plants to convert low energy nitrogen sources that most other organisms avoid like NO3^−^ into organic nitrogen compounds. This confers an evolutionary advantage to C_3_ plants, which remain dominant among plant species [3]. Under the current rapid surge in atmospheric CO_2_ level, a condition which slows photorespiration, C_3_ species using NO_3_^−^ as a nitrogen source suffer most from decreased organic nitrogen production [179,183]. N2-fixing legumes and C4 plants with CO_2_ concentrating mechanisms, are more resilient to changes in CO_2_ [174] because their inorganic nitrogen acquisition does not depend on photorespiration. 

The use of NH_4_^+^-based nitrogen fertilizer and breeding for genotypes with improved NH_4_^+^ assimilation and tolerance may offer a solution for sustaining plant protein levels under future CO_2_-enriched atmospheres [184,185]. Biological Nitrification Inhibitors (BNI), which allow certain plant species to regulate their rhizosphere pools of inorganic nitrogen by releasing root exudates that specifically inhibit nitrifying bacteria that convert NH_4_^+^ into NO_3_^−^, may be beneficial [186]. Application of artificial BNI chemicals or incorporation of this trait into new cultivars may enhance crop growth under NH_4_^+^ nutrition [185,187]. 

Surprisingly, given the chemical differences between NO_3_^−^ and NH_4_^+^, relatively little is known about how various nitrogen supplies shape crop adaptation and yield in a field setting (Table 3; see discussion below). The ability of the current germplasm to employ a specific nitrogen form as their predominant nitrogen source and maintain productivity at elevated CO_2_ levels thus remains an open question. 

### 5.3. Little Information about Natural Genetic Variation and Genome-Wide Interactions Limits Breeding Applications

Despite an increasing understanding of physiological adaptation of roots and shoot to nitrogen supply [122], less is known about genetic adaptations [188]. Recent advances in genetic approaches greatly facilitate the identification of genes responsible for specific physiological traits. Of particular interest are Genome Wide Association Studies (GWAS) that use extensive sets of molecular markers to explore genetic variation resulting from historical recombinant events and from adaptation to changes in environmental conditions over evolutionary time [189]. Genetic architecture of traits also strongly influences GWAS robustness such that traits with rare alleles are more difficult to identify [190,191]. 

To date, a combination of GWAS and linkage mapping have identified many loci that underlie nitrogen responses of agricultural crops [192,193]. Importantly, GWAS enables deeper understanding of how environments may have shaped crop adaptation [194]. As such, natural variation of functional alleles can help inform breeding applications to achieve a better match between genotype and location [195]. Haplotype analyses in global germplasm quantifies allelic frequency of different breeding and natural subpopulations [196] and can offer practical strategies in breeding programs [197]. 

Selection pressures that vary during the history of crop domestication or with local limiting growth factors [198] provide insights into crop evolution and adaptation. More commonly, studies focus on differences between major subpopulations and how selection drives divergence or convergence between them. For example, divergence between the indica and japonica subpopulations of rice can be accounted by variations in key nitrogen metabolism genes like *NPF6.5* [57] or *NR2* [72]. Around 8% of the rice genome covering major nitrogen metabolism genes appear to be under selection including *AMT1.1*, *NRT2.3*, *NAR2.2*, *NIR1*, *GS1;2*, and *GS1;3* [199]. Unfortunately, information is limited about the natural variation in candidate genes that enhance yield and the extent to which they have been under selection (Table 3). For instance, *OsNPF6.1*, which was identified through GWAS and functions under low nitrogen supply to increase NO_3_^−^ uptake, is considered a rare allele, because it is present in less than 10% of cultivated varieties [55]. The absence or presence of a functional allele from diverse geographic regions may reflect adaptation to a particular soil nitrogen pool.

Apart from a few studies [83,86], we have limited information on the extent to which natural soil nitrogen availability shapes crop adaptation and, in turn, on subsequent responses to external nitrogen fertilizers in agricultural production systems. For example, *Ghd7* allelic variation also correlates with soil nitrogen deposition rates [86]. Likewise, rice *OsTCP19*, which was identified through a GWAS on tiller responsiveness to nitrogen availability, has a functional allele frequency that is correlated with soil nitrogen concentration, and the nitrogen-responsive genotypes are more common in regions with low nitrogen concentrations [83]. Extensive networks of genes interact to sense and signal perception of nitrogen, especially NO_3_^−^ [106]. Interestingly, expression of *OsTCP19* follows changes in NO_3_^−^, but not NH_4_^+^ [83]. Overall, evidence is insufficient to conclude whether crops like wheat and rice, which have been exposed over the long term to certain nitrogen forms, show adaptation to a particular form. This information is vital for applying robust breeding strategies to improve future crops.

Genetic × environment interactions and expression patterns contingent upon growth conditions influence phenotypic plasticity [200,201], even when the same genes are being modified. Specifically, some genes may only be beneficial in certain environments or may even have detrimental pleiotropic effects in others. Field trials indicate that yield enhancement is highly dependent on growing conditions. For example, overexpression of *ASN1* enhanced rice grain yield in pot experiments under limited nitrogen supply, but had no observable effect under sufficient nitrogen supply in the field [73]. In sites with a longer growing season, *DREB1C* transgenic plants exhibited a much higher yield boost compared to wildtypes [95]. With more advanced molecular breeding and transgenic approaches, promoters inducible in specific tissues or by desirable environmental triggers could perhaps mitigate such issues [12,121]. Precision genome editing methods, like the CRISPR-Cas9 system, facilitate genetic modifications at multiple target tissues, developmental times, and traits all at once without the introduction of foreign genetic materials [202]. Furthermore, advanced GWAS pipelines allow more explicit consideration of environmental variations to quantify plasticity and predict phenotype in a particular environment [203]. Better understanding of crop genetics, yield components, and their responses to the environment should bridge the gap between improved nitrogen metabolism and yield improvement. 

Epistatic interactions further complicate breeding for candidate genes in different genetic backgrounds [204]. Gene or trait stacking based on our current understanding of each individual gene, protein, or process have had limited success to date, perhaps because of too little understanding of the complex regulatory network [205]. For example, the introduction of the grain protein content *NAM-B1* transcription factor functional allele, which is generally absent from modern varieties, has only minimal influence on yield, but enhances grain nitrogen and protein content significantly across a wide range of environments [26,206,207]. A meta-analysis across 40 environments showed that 19% of bread wheat genotypes with *NAM-B1* functional alleles exhibit yield enhancement [207], suggesting that the global germplasm still has genetic yield potential. Furthermore, combining multiple nitrogen metabolism genes in the pathway, for example *NR2* and *NPF6.5* [72], or *AMT1;2* with *GOGAT* [69] offers greater chance of yield enhancement than modulating individual genes alone. These observations argue for manipulating either gene networks with multiple genes of relatively small effects or transcription factors that affect several genes and processes at the same time. Further understanding of system biology, especially underlying nitrogen metabolism, should prove useful in guiding such manipulations.

## 6. Concluding Remarks

Both yield and nitrogen metabolism pathways are complex traits with multiple layers of genetic control. While actual farm yield has increased in some regions of the world, increases in cereal potential yield—the scenario with no limitation on crop growth—have fallen down to below 1% annually [7]. We urgently need to apply new breeding strategies that accelerate genetic gains to meet the demands of our growing human population. 

Here, we considered NUE on the basis of grain and total biomass production per unit of nitrogen applied or assimilated. Improvement of nitrogen acquisition, however, does not always translate into higher yields. For example, overexpression of transporter *NPF7.4* resulted in higher NO_3_^−^ uptake, lower NO_3_^−^ accumulation, but higher tissue amino acid concentration, indicating improved nitrogen assimilation; nevertheless, such enhanced nitrogen acquisition decreased biomass and grain production [58]. Knocking out *Lysine-Histidine-type Transporter 1* (*LHT1*), which transports amino acid, helped improve grain nutritional quality at maturity, but at the expense of vegetative biomass, grain weight, and germination rate [208,209]. Henceforth, defining and setting NUE as breeding targets to lower agricultural nitrogen inputs must take into account grain protein content [210,211]. These efforts are prime candidates for improving grain nutritional values. Therefore, if we define NUE as the amount of organic nitrogen that ends up in the consumable grains per nitrogen applied, these genes are worthy of consideration. 

Breeding strategies that focus concurrently on both carbon and nitrogen assimilation also offer an opportunity to break the longstanding antagonistic relationship between grain biomass and protein concentration [212] that hampers genetic gains in yield over time. Genetic solutions are needed because management practices like applications of nitrogen fertilizers at booting stage to meet grain nitrogen demand can only partially alleviate this negative relationship at the field level [213]. Control of *NGR5* that uncouples yield components from nitrogen-dependent responses [82], or *GRF4* that breaks the tie between dwarfism-induced yield improvement and reduced nitrogen assimilation [42], establish the possibility of maximizing both yield and NUE at the same time. Genomic selection is theoretically feasible and genomic breeding tools are becoming readily available for breeders to target both sets of traits simultaneously [197,214].

## 7. Conclusions

This review highlights achievements in manipulating the genetics underlying nitrogen metabolism pathways to enhance yield of rice and wheat, focusing on relationships between yield components and crop nitrogen use during growth. Further fundamental understanding of ortholog genes between species, how different forms of nitrogen influence growth and development, and natural variation of desirable traits responsive to nitrogen should prove useful in achieving higher crop yields. Hopefully, continuous, albeit slow, progress on genetic gain in crop nitrogen assimilation and yield over time can fulfill the yield gap needed to feed our global community.

## Figures and Tables

**Table 1:** Average contribution of wheat and rice to daily protein intake between 2010 and 2019 [19].

Region		Protein Supply in FAO’s Food Balance Sheet (g per Capita per Day)					
		Total Intake	From Wheat	%	From Rice	%	% Wheat and Rice
World		81.39	16.28	20.00	10.08	12.38	32.38
Africa	Eastern Africa	60.06	6.31	10.50	3.35	5.58	16.09
	Middle Africa	45.17	3.50	7.75	2.39	5.29	13.04
	Northern Africa	93.05	35.33	37.97	3.45	3.71	41.68
	Southern Africa	78.91	13.76	17.44	2.77	3.51	20.95
	Western Africa	63.46	5.02	7.92	7.87	12.40	20.32
America	Caribbean	67.31	9.40	13.97	9.37	13.93	27.89
	Central America	83.73	6.86	8.19	2.05	2.45	10.64
	Northern America	110.57	19.31	17.46	1.44	1.30	18.76
	South America	86.53	11.89	13.74	5.53	6.40	20.13
Asia	Central Asia	91.29	35.14	38.49	1.26	1.38	39.87
	Eastern Asia	98.35	17.29	17.58	14.89	15.14	32.72
	South-eastern Asia	69.05	4.82	6.99	23.52	34.07	41.05
	Southern Asia	62.71	16.48	26.29	13.09	20.88	47.17
	Western Asia	87.67	34.33	39.16	3.72	4.24	43.40
Europe	Eastern Europe	97.68	28.77	29.45	0.69	0.70	30.15
	Northern Europe	106.60	24.27	22.76	1.13	1.06	23.83
	Southern Europe	104.59	26.91	25.73	1.25	1.19	26.92
	Western Europe	105.43	23.54	22.33	0.83	0.79	23.12
Oceania	Australia/New Zealand	106.23	18.93	17.82	1.73	1.63	19.45
	Melanesia	65.03	8.40	12.92	3.74	5.75	18.67
	Micronesia	71.28	10.86	15.23	12.60	17.68	32.91
	Polynesia	92.76	14.25	15.36	4.17	4.50	19.86

**Table 2:** Breeding applications of nitrogen metabolism genes in rice and wheat that were proven successful in improving yield.

#	Gene		Ref	Species	Breeding Application	Yield Component Improvement					
						Yield	Tillering	Grain weight	Grain number	Biomass NUE	Other/Note
1	*Nitrate transporter 1/Peptide transporter Family 6.1*	*NPF6.1*	[55]	Rice	Overexpression	✓	✓ *				* Effective panicle number. No data on grain number and weight.
2	*Nitrate transporter 1/Peptide transporter Family 6.3*	*NPF6.3 (NRT1.1A)*	[56]	Rice	Overexpression	✓				✓	Shortened maturation time
3	*Nitrate transporter 1/Peptide transporter Family 6.5*	*NPF6.5 (NRT1.1B)*	[57]	Rice	Near-isogenic line, Transgenic japonica with indica variant	✓	✓			✓	
4	*Nitrate transporter 1/Peptide transporter Family 7.1*	*NPF7.1*	[58]	Rice	Overexpression	✓	✓		✓	✓	
5	*Nitrate transporter 1/Peptide transporter Family 7.1*	*NPF7.2*	[59]	Rice	Overexpression	✓	✓				Increased root length, root number, root biomass
6	*Nitrate transporter 1/Peptide transporter Family 7.4*	*NPF7.4*	[58]	Rice	CRISPR/Cas9 mutant	✓	✓		✓	✓	
7	*Nitrate transporter 1/Peptide transporter Family 7.7*	*NPF7.7*	[60]	Rice	Overexpression	✓ *	✓		✓	✓	* Yield presented as g grain/g N. Larger panicle, Higher N content, but not amino acid suggests N accumulation
8	*Nitrate transporter 1/Peptide transporter Family 8.20*	*NPF8.20 (PTR9)*	[61]	Rice	Overexpression	✓	✓	✓	✓	✓	Highest improvement at low N
9	*High-affinity nitrate transporter 2.1*	*NRT2.1*	[62,63]	Rice	Overexpression	✓					Increased Mn accumulation
10	*High-affinity nitrate transporter 2.3b*	*NRT2.3b*	[64]	Rice	Overexpression	✓				✓	
11	*High-affinity nitrate transporter-activating protein 2.1*	*NAR2.1*	[63,65,66,67]	Rice	Overexpression, Transgenic with native promoter	✓				✓	
12	*Ammonium transporter 1;1*	*AMT1;1*	[68]	Rice	Overexpression	✓					
13	*Ammonium transporter 1;2*	*AMT1;2*	[69]	Rice	Double activation mutants with *GOGAT1*	✓				✓	
14	*Glutamate synthase 1*	*GOGAT1*	[69]	Rice	Double activation mutants with *AMT1;2*	✓				✓	
15	*Glutamine synthetase 1*	*GS1*	[70]	Rice	Overexpression	✓	✓		✓		
16	*Glutamine synthetase 2*	*GS2*	[70,71]	Wheat, Rice	Overexpression	✓		✓	✓		
17	*Nitrate reductase 2*	*NR2*	[72]	Rice	Transgenic japonica with indica variant	✓	✓			✓	
18	*Asparagine synthetase 1*	*ASN1*	[73]	Rice	Overexpression	✓ *				✓	* Yield increases only at low N
19	*Amino acid permease 1*	*AAP1*	[74]	Rice	Overexpression	✓	✓		✓		
20	*Amino acid permease 3*	*AAP3*	[75]	Rice	RNAi	✓	✓				
21	*Amino acid permease 4*	*AAP4*	[76]	Rice	Overexpression	✓	✓				
22	*Amino acid permease 5*	*AAP5*	[77]	Rice	RNAi	✓	✓				
23	*Nodule Inception-Like protein 1*	*NLP1*	[78]	Rice	Overexpression	✓				✓	
24	*Nodule Inception-Like protein 3*	*NLP3*	[79]	Rice	Overexpression	✓				✓	
25	*Nodule Inception-Like protein 4*	*NLP4*	[80,81]	Rice	Overexpression, Quadrupling the promoter of *NiR*	✓	✓				
26	*Growth-Regulating Factor 4*	*GRF4*	[42]	Rice, Wheat	Overexpression	✓	✓ *	✓	✓ *	✓	* Depending on genetic background; Tiller number not changed in *dep1*-driven dwarfism and in *NJ6-sd1*. Grain number not changed in *NJ6-sd1*
27	*Nitrogen-mediated tiller Growth Response 5*	*NGR5*	[82]	Rice	Overexpression	✓	✓	?	?	?	Did not show changes in yield components apart from tiller number for *NGR5* transgenic plants.
28	*Teosinte branched1, Cycloidea, Proliferating cell factor 19*	*TCP19*	[83]	Rice	Introgression	✓	✓		✓		
*29*	*NAM/ATAF1/2/CUC2 42*	*NAC42*	[55]	Rice	See *NPF6.1*						
*30*	*NAM/ATAF1/2/CUC2 2-5A*	*NAC2-5A*	[84]	Wheat	Overexpression	✓				✓	Increased root growth
31	*Basic Leucine Zipper 60*	*bZIP60*	[85]	Wheat	RNAi	✓			✓		Increased N uptake and NADH-GOGAT
32	*Grain Number, Plant Height, and Heading Date 7*	*Ghd7*	[86]	Rice	Overexpression	✓				✓	
33	Abnormal cytokinin response1 REpressor 1	ARE1	[87,88]	Rice, Wheat	Loss-of-function, CRISPR/Cas9 mutant	✓					Delayed senescence
34	*N-mediated heading date 1*	*Nhd1*	[89,90]	Rice	Knockout mutant	✓	✓			✓	Increased N uptake
35	*Dense and Erect Panicle 1*	*DEP1*	[91,92,93]	Rice	Loss-of-function, gain of function mutant	✓			✓		
36	*Dull Nitrogen Response 1*	*DNR1*	[94]	Rice	Loss-of-function mutant	✓			✓		Lower tiller number. Total biomass/grain weight not reported.
37	*Dehydration-Responsive Element-Binding Protein 1C*	*DREB1C*	[95]	Rice, Wheat	Overexpression	✓		✓	✓	✓	Early flowering, Higher photosynthesis, Higher N uptake, Higher harvest index
38	*Nuclear Factor Y A-B1*	*NFYA-B1*	[96]	Wheat	Overexpression	✓	✓				Higher N uptake, Higher grain N content, More lateral root growth
*39*	*Rice Dof Daily fluctuations 1*	*RDD1*	[97]	Rice	Overexpression	✓		✓	✓		Higher harvest index, Altered uptake of multiple nutrients, Early flowering
*40*	*MicroRNA 396*	MIR396	[98]	Rice	Knock-out mutant	✓		✓	✓	✓	15% yield increases under low N, Larger panicle

**Table 3:** Functions, selection, and effects on nitrogen acquisition of nitrogen metabolism genes in rice and wheat that were proven successful in improving yield. Empty cells indicate the lack of information.

#	Gene	Ref	Function	Natural Variation and Selection	Effects on NH_4_^+^	Effects on NO_3_^−^
1	*NPF6.1*	[55]	NO_3_^−^ uptake; Must be activated by *NAC42* transcriptional factor	Rare allele absent in 90.3% of rice varieties		Increased uptake/concentration
2	*NPF6.3 (NRT1.1A)*	[56]	Upregulate N utilization and flowering genes			
3	*NPF6.5 (NRT1.1B)*	[57,158]	NO_3_^−^ uptake, transporter; Upregulate NO_3_^−^ responsive genes	Directional positive selection. Indica has a functional variant.		
4	*NPF7.1*	[58]	Determine axillary bud outgrowth; NO_3_^−^ uptake			Increased influx/concentration
5	*NPF7.2*	[59]	Upregulate genes in cytokinin pathway, thereby increasing cytokinin concentration; Downregulate genes in strigolactone biosynthesis, perception and signaling pathway, thereby reducing suppression on tillering.			Increased influx/concentration
6	*NPF7.4*	[58]	Determine axillary bud outgrowth, NO_3_^−^ uptake			Increased influx/concentration
7	*NPF7.7*	[60]	Two splicing variants transport distinct N forms; Upregulate *GS1.2*, *NPF6.5*; Downregulate *Fine Culm1 (FC1)*, *Dwarf3 (D3)* to regulate tillering.		Increased influx/concentration for both variant, Higher for variant 2	Increased influx/concentration for variant 1 only
8	*NPF8.20 (PTR9)*	[61,164]	Upregulate *GS*, *AMT1;2*; Increase lateral root density		Increased uptake	
9	*NRT2.1*	[62,63]	High affinity NO_3_^−^ transporter; Responsive only to NO_3_^−^; Interact with *NAR2.1*.			
10	*NRT2.3b*	[64,165]	Buffering pH; NO_3_^−^ uptake; Increase NH_4_^+^ uptake even though it does not transport NH_4_^+^	Under selection. Expression ratio of two variants correlated with vegetative N content.		Increased uptake
11	*NAR2* *.1*	[63,65,66,67,165,166]	NO_3_^−^ uptake, interacting with *NRT2.1, NRT2.2, NRT2.3a*			Increased uptake
12	*AMT1;1*	[68,167,168]	NH_4_^+^ uptake under low and high NH_4_^+^ conditions; N/K homeostasis		Increased uptake	
13	*AMT1;2*	[69,167]	NH_4_^+^ uptake and remobilization			
14	*GOGAT1*	[69]	NH_4_^+^ uptake and remobilization			
15	*GS1*	[70,169]	Coordinate N metabolic balance and remobilization; Confer tolerance to abiotic stresses; Must be expressed concurrently with *GS2.*			
16	*GS2*	[70,71]	Increase root N uptake before and after flowering, N mobilization and N harvest index; Prolong leaf photosynthesis post-anthesis; Increase expression of *NRT2.1* and *NPF 6.3*; In rice, must be expressed concurrently with GS1.	2 haplotypes in A genome		
17	*NR2*	[72]	Encode *NADH/NADPH-dependent NO_3_^−^ reductase*; Interact with *NPF6.5* to control NO_3_^−^ uptake	Diverged between indica and japonica.		Increased uptake
18	*ASN1*	[73]	Upregulate A*MT1;1, AMT1;2, AMT1;3, GS1;1, NADH-GOGAT1*		Increased uptake	
19	*AAP1*	[74,170]	Facilitate amino acid transportation to reproductive organs			
20	*AAP3*	[75]	Reduced expression promotes tiller bud elongation, relatively more than formation, via balancing basic and neutral amino acid to maintain higher cytokinin	25 haplotypes. Promoter sequence differs between indica and japonica		
21	*AAP4*	[76]	Higher expression in indica produce more tiller and grain yield	5 haplotypes. Promoter sequence differs between indica and japonica		
22	*AAP5*	[77]	Reduced expression regulate tiller bud via balancing basic and neutral amino acid to maintain higher cytokinin	11 promoter variants. Sequence differs between indica and japonica		
23	*NLP1*	[78]	Regulate transcription of N related genes and transcriptional factors (both NO_3_^−^ and NH_4_^+^)			
24	*NLP3*	[79]	Bind to NO_3_^−^-responsive cis-elements in promoters of N uptake and assimilation genes; Overlaps with *NLP1* and *NLP4*			
25	*NLP4*	[80,81]	Regulate expression of known N genes by binding to NO_3_^−^- responsive cis-element in promoter, Activate *NiR*	2 haplotypes		
26	*GRF4*	[42]	Counteracts DELLA to promote N assimilation both NO_3_^−^ and NH_4_^+^; Upregulate expression of *AMT1.1, GS1.2, GS2, NADH-GOGAT2, NRT1.1B, NRT2.3a, NPF2.4, NIA1, NIA3, NiR1* and genes related to photosynthesis, C metabolism and cell division to maintain stable C:N ratio; Highest expression at low N;	3 haplotypes. Haplotype B has highest yield. Absent from elite varieties.	Increased uptake	Increased uptake
27	*NGR5*	[82]	Recruit PRC2 upon increased N supply to promote H3K27me3 modification that represses shoot branching inhibitory genes; DELLA proteins stabilize *NGR5* and sustain tiller promotion by competitively inhibiting gibberillin-driven destruction of *NGR5*.	5 haplotypes. Haplotype 2 contains a functional variant.		
28	*TCP19*	[83]	Represses tiller promoting gene *DLT*, the product of which can interact directly with *NGR5*, to negatively control cellular bud growth			
*29*	*NAC42*	[55]	Activate *NPF6.1*			
*30*	*NAC2-5A*	[84]	Regulate expression of NO_3_^−^ transporter and *GS*			Increased uptake
31	*bZIP60*	[85]	Negative regulation on *NADH-GOGAT*			
32	*Ghd7*	[86,171]	Repress *ARE1* to positively regulate N utilization	At least 10 alleic variants. Alleic frequency correlates with N deposition rate.		
33	*ARE1*	[87,88]	Suppress *Fd-GOGAT*	3 haplotypes in promoter sequence. Under selection.		
34	*Nhd1*	[89,90]	Activate Hd3a for flowering time; Control negative feedback on N assimilation (loss-of-function increases Fd-GOGAT and LHT1 activities); Activate *AMT1;3, NRT2.4*	5 haplotypes. Similar between indica and japonica. Variation in promoter associated with nitrogen.		
35	*DEP1*	[91,92,93,172]	Interact to reduce heterotrimeric G-protein α-subunit 1 (*RGA1*) or enhance β-subunit 1 (*RGB1*) to inhibit N responses; Promote aerenchyma formation; Upregulate *GS/GOGAT*	Under selection during japonica domestication.		
36	*DNR1*	[94]	Negative regulator of auxin-regulated N metabolism; N supply lowers *DNR1*, thereby inducing Auxin Response Factors to upregulate *NPF6.5, NRT2.3a, NPF2.4,* and *NIA2*.	3 haplotypes. Haplotype A specific to indica is absent from japonica.		Increased uptake
37	*DREB1C*	[95]	Regulate NT2, NRT2.4, NPF6.5	3 haplotypes in promoter sequence. Haplotype 3 superior.		Increased uptake
38	*NFYA-B1*	[96]	Control root development and N, P usage			Increased uptake
39	*RDD1*	[97]	Upregulate *AMT1;3, GS1;1;* Uptake of N, P, K, Na, Mg, Cl, S, Ca	Highly conserved in wild rice relatives.	Increased uptake/accumulation	Increased uptake
40	*MIR396*	[98]	Only isoform e and f; Upregulate GRF4, GRF6, GRF8, NIR1, NIR2, GOGAT2, GS1.2, AAPs			

## Data Availability

No new data were created in this study. Data sharing is not applicable to this article.
